# Sustainable Recycling of Lithium-Ion Battery Cathodes: Life Cycle Assessment, Technologies, and Economic Insights

**DOI:** 10.3390/nano15161283

**Published:** 2025-08-20

**Authors:** Dongjie Pang, Haoyu Wang, Yimin Zeng, Xue Han, Ying Zheng

**Affiliations:** 1 Department of Chemical and Biochemical Engineering, Western University, London, ON N6A 5B9, Canada; dpang28@uwo.ca (D.P.); hwang928@uwo.ca (H.W.); 2 CanmetMATERIALS, NRCan, Hamilton, ON L8P 0A5, Canada; yimin.zeng@nrcan-rncan.gc.ca

**Keywords:** lithium-ion battery, cathode material recycling, life cycle assessment, technoeconomic analysis

## Abstract

Rapid growth of electric vehicles has increased demand for lithium-ion batteries (LIBs), raising concerns regarding their end-of-life management. This study comprehensively evaluates the closed-loop recycling of cathode materials from spent LIBs by integrating life cycle assessment (LCA), technoeconomic analysis, and technological comparison. Typical approaches—including pyrometallurgy, hydrometallurgy, and other processes such as organic acid leaching and in situ reduction roasting—are systematically reviewed. While pyrometallurgy offers scalability, it is hindered by high energy consumption and excessive greenhouse gas emissions. Hydrometallurgy achieves higher metal recovery rates with better environmental performance but requires complex chemical and wastewater management. Emerging methods and regeneration techniques such as co-precipitation and sol–gel synthesis demonstrate potential for high-purity material recovery and circular manufacturing. LCA results confirm that recycling significantly reduces GHG emissions, especially for high-nickel cathode chemistry. However, the environmental benefits are affected by upstream factors such as collection, disassembly, and logistics. Technoeconomic simulations show that profitability is strongly influenced by battery composition, regional cost structures, and collection rates. The study highlights the necessity of harmonized LCA boundaries, process optimization, and supportive policy frameworks to scale environmentally and economically sustainable LIB recycling, ensuring long-term supply security for critical battery materials.

## 1. Introduction

With the accelerated global shift toward low-carbon transportation, electric vehicles (EVs) have become a major driver of lithium-ion battery (LIB) demand [1]. In recent years, EV sales have demonstrated consistent growth. By 2030, EVs are anticipated to constitute 42% of the global vehicle market, and the share is projected to increase further, leading to the production of over 60 million EVs by 2040 [2,3,4]. This significantly accelerates the consumption and eventual disposal of LIBs [5]. According to the requirements of EV manufacturers and EV safety, a battery will be retired when its capacity decays to less than 80% of the initial capacity [6].

**Figure 1:**
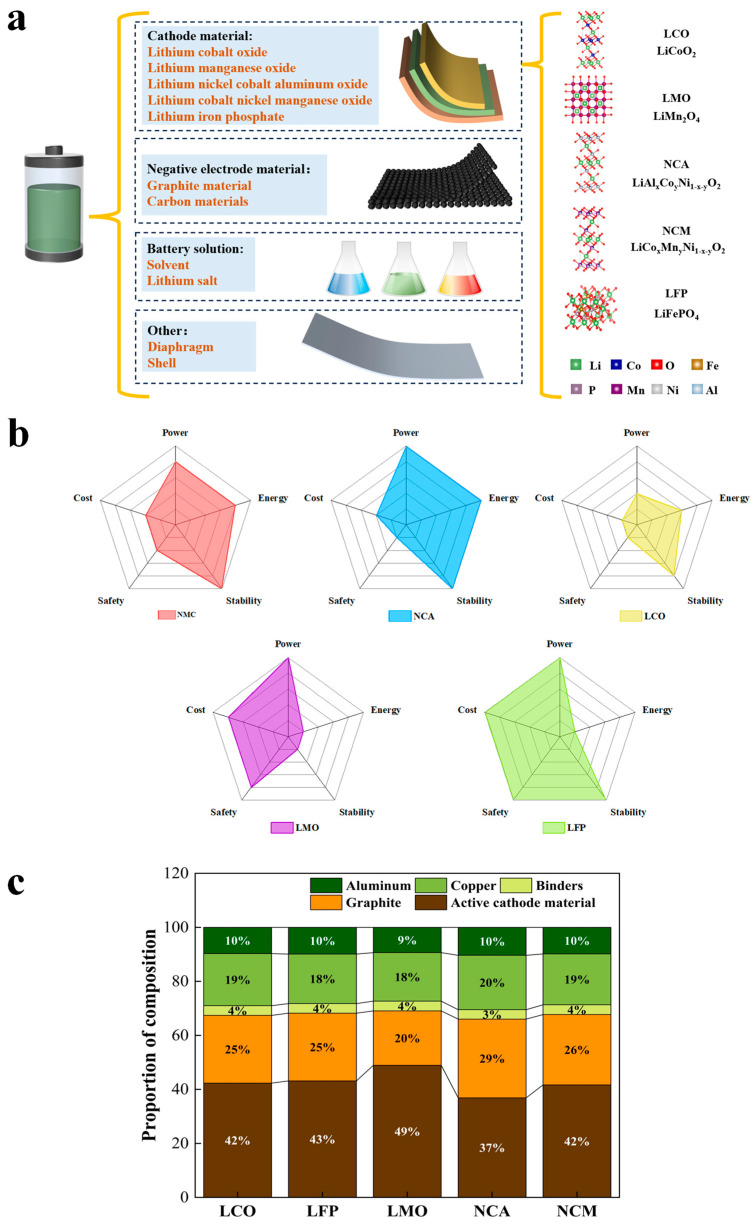
(**a**) Main components of automotive power batteries and the positive crystal structures of common LIB cathode materials. (**b**) Radar chart of performance comparison for various types of LIB cathode materials. (**c**) Typical compositions of various types of batteries [13].

LIBs typically consist of a cathode, an anode, an electrolyte, a separator (diaphragm), and a shell. Critical cathode minerals like lithium, cobalt, and nickel must be recycled, along with graphite from anode to ensure resource sustainability. However, this review will focus on the recycling of critical minerals from cathodes. The compositions of typical LIB cathode materials and their strengths and limitations are shown in Figure 1b. Nickel-manganese-cobalt (NMC) batteries demonstrate a balanced performance across energy density, power capability, cost, and safety, making them the most widely used chemistry in current electric vehicle (EV) applications. Common commercial compositions such as NMC111, NMC622, and NMC811 vary in nickel content, where increasing nickel improves capacity but reduces thermal stability [7]. Nickel-cobalt-aluminum (NCA) batteries, by contrast, offer superior energy and power densities and are commonly deployed in high-performance EVs. However, their adoption is limited due to higher costs and lower thermal stability. Despite these drawbacks, they provide higher average voltages, resulting in better energy density performance than lithium iron phosphate (LFP) batteries [8,9]. LFP batteries are increasingly favored for mid- and low-range EVs due to their chemical stability, low cost, and improving energy performance. Although limited in energy density compared to NMC and NCA, LFP batteries offer excellent safety and thermal stability, with thermal decomposition temperatures reaching approximately 700 °C—substantially higher than the 200–300 °C range for NMC or NCA materials [10,11]. However, recent studies have shown that thermal runaway is primarily influenced by anode stability, SEI layer degradation, and lithium plating under abnormal charging, whereas the contribution of cathode material is minor [12]. Lithium cobalt oxide (LCO) offers high energy density but suffers from high cost and safety issues due to elevated cobalt content, while lithium manganese oxide (LMO) provides cost-effectiveness and safety at the expense of lower energy density and lifespan, making it suitable for hybrid or short-range EVs [13]. However, recent studies have shown that thermal runaway is primarily influenced by anode stability, SEI layer degradation, and lithium plating under abnormal charging, whereas the contribution of cathode material is minor [14]. Figure 1c illustrates the ratios of major components of various battery types. These compositional differences significantly influence key battery characteristics, including energy density, lifespan, and charging speed, highlighting the trade-offs associated with material selection for EV applications. To highlight the economic motivation for recycling, Table 1 presents market prices of the critical minerals recycled from spent batteries.

The LIB recycling industry is currently dominated by pyrometallurgical and hydrometallurgical technologies, each facing distinct economic and environmental challenges. Pyrometallurgy enables efficient metal recovery via high-temperature treatment but entails high energy consumption and generates hazardous emissions, including metal-laden slag and gases. The relatively low purity of recovered metals also necessitates additional refining, increasing costs and environmental burden. Hydrometallurgy, while offering higher recovery selectivity, incurs 30–40% of non-material costs from auxiliary reagents and faces chemical price volatility. It also produces wastewater and solid residues containing residual heavy metals and reagents, posing environmental risks. In addition, both approaches are constrained by technical bottlenecks such as incomplete binder removal, electrolyte decomposition, and residual impurities. This paper aims to bridge this gap by integrating life cycle assessment (LCA), economic modeling, and technological comparison to evaluate and optimize the sustainability of LIB recycling processes.

## 2. Cathode Materials in Lithium Battery Recycling Process and Technology

Global recycling technologies for lithium-ion power batteries have reached a high level of maturity, typically involving three main stages: pretreatment, dismantling and separation, and material recovery. Material recovery mainly involves pyrometallurgical and hydrometallurgical techniques [17]. Different companies worldwide have adopted various recycling technologies to deal with spent lithium-ion power batteries and obtain different main recycled products, as shown in Table 2.

Recycling not only mitigates the environmental risks posed by battery waste but also facilitates the recovery of critical metals such as lithium, cobalt, nickel, and manganese Table 3 provides an overview of key stages and technologies for LIB cathode materials recycling, emphasizing their respective processes, advantages, and limitations. The recycling process begins with pretreatment, where spent batteries undergo discharge and deactivation to ensure safe handling during dismantling. Discharge methods include chemical immersion in NaCl solutions and low-temperature freezing [29]. Following pretreatment, the batteries are disassembled to separate key components, such as cathode materials, aluminum and copper foils, and casings. Mechanical separation, which includes dismantling, crushing, and screening, is commonly used for large-scale operations due to its simplicity and independence from chemical reagents [30]. Traditional recycling routes for cathode materials primarily involve pyrometallurgy and hydrometallurgy. Pyrometallurgy employs high-temperature smelting (> 1000 °C) to recover metals in alloy form, but this method is energy-intensive and releases toxic gases such as HF and SO_2_, posing environmental and safety challenges [31]. In contrast, hydrometallurgy uses strong inorganic acids (e.g., H_2_SO_4_) to leach metals, often achieving extraction efficiencies above 95%. However, it also generates large volumes of acidic wastewater and requires multi-step purification processes, increasing treatment complexity [32]. Emerging alternatives like organic acid leaching offer reduced environmental hazards and equipment corrosion [33]. Organic acids like citric acid and acetic acid demonstrate comparable metal recovery rates but require further optimization to simplify their complex mechanisms. Ammonia leaching has garnered attention due to its simplified separation processes and lower industrial costs. However, it generates ammonium-based waste that must be properly disposed of [34]. Once metal ions are recovered from the leachate, purification and material synthesis take place through methods such as solvent extraction, chemical precipitation, or advanced techniques like the sol–gel process.

To enable effective metal recovery, separating the electrode materials obtained during the dismantling process, thereby isolating the active materials from their current collectors, is essential. Cathode active materials firmly adhered to aluminum foil using organic binders such as polyvinylidene fluoride (PVDF). Hence, efficiently separating cathode materials from aluminum foil is a critical prerequisite for the recycling of lithium-ion batteries [35]. Standard separation methods include thermal treatment, organic solvent extraction, and alkaline dissolution. Selecting the right thermal treatment temperatures allows for the detachment of cathode materials from the aluminum foil while also removing organic impurities through thermal processing [36]. PVDF begins decomposition at temperatures between 350 and 450 °C and is fully decomposed by 600 °C. At this elevated temperature, conductive agents are also oxidized and eliminated, yielding cathode materials of relatively high purity [37]. The organic solvent dissolution method entails the use of solvents with high solubility for PVDF to effectively remove PVDF binders. Commonly employed organic solvents include N-methylpyrrolidone (NMP), N,N-dimethylformamide (DMF), and dimethylacetamide (DMAC). This method achieves a high separation efficiency but is constrained by its lengthy processing time [38].

### 2.1. Pyrometallurgical-Based Recycling of Cathode Materials

The pyrometallurgical recycling process employs high-temperature smelting to recover valuable metals from spent lithium-ion batteries in the form of alloys or metal compounds [39]. This approach is advantageous due to its operational simplicity and capacity for large-scale processing, as it generally does not require pretreatment of battery materials. Additionally, it eliminates the generation of acidic or alkaline waste, contributing to its significant role in the battery recycling market [40]. Depending on the reagents employed, pyrometallurgical recycling can be divided into slag-based reduction smelting, carbothermal reduction (CTR), and salt-assisted roasting (SAR), as summarized in Table 4.

Slag-based reduction smelting utilizes a basic slag system to induce physical and chemical changes at high temperatures, facilitating the enrichment and precipitation of valuable metals [50]. Organic components within the batteries can act as reducing agents, promoting the formation of alloy compounds containing metals such as Cu, Co, Fe, and Ni [51]. This method benefits from its capacity to handle mixed materials and recover a wide range of metals. However, it may result in the loss of lithium into the slag phase, limiting its effectiveness for lithium recovery.

Carbothermal reduction (CTR) is often employed to improve lithium recovery by selectively converting insoluble lithium compounds into water-soluble lithium species [52]. It uses carbon sources such as charcoal, graphite, or coke as reducing agents and operates at relatively lower temperatures (650–1000 °C) compared to traditional high-temperature smelting. During approximately one hour of heating, cathode materials are reduced to metals and transition metal oxides, while lithium is transformed into easily recoverable compounds such as Li_2_CO_3_ or Li_2_O, thereby preventing its loss in slag as observed in conventional smelting [53,54]. This process is particularly advantageous for its efficiency in lithium recovery and its lower energy requirements. The CTR process involves a series of chemical reactions, including the overall reduction of LiCoO_2_ (Equation (1)) and the conversion of CoO to metallic Co (Equations (2)–(3)). For LiNiO_2_-based cathodes, CTR proceeds through decomposition into NiO and Li_2_O (Equation (4)), followed by NiO reduction to Ni (Equations (5)–(6)), ultimately facilitating lithium recovery as Li_2_CO_3_. In the case of LiMn_2_O_4_, CTR follows a pathway of stepwise decomposition into MnO and other intermediates (Equations (7)–(8)), coupled with the reduction of manganese oxides by carbon or CO gas (Equation (9)). These reactions demonstrate the versatility of CTR in recovering both lithium and transition metals efficiently.

(1) $$4\mathrm{LiCo}O_{2}+3C\to 2Li_{2}CO_{3}+4\mathrm{Co}+CO_{2}$$

(2) $$\mathrm{CoO}+CO_{\left(g\right)}\to \mathrm{Co}+CO_{2\left(g\right)}$$

(3) $$2\mathrm{CoO}+C\to 2\mathrm{Co}+CO_{2\left(g\right)}$$

(4) $$4\mathrm{LiNi}O_{2}+3C\to 2Li_{2}CO_{3}+4\mathrm{Ni}+CO_{2\left(g\right)}$$

(5) $$2\mathrm{NiO}+C\to 2\mathrm{Ni}+CO_{2\left(g\right)}$$

(6) $$\mathrm{NiO}+C\to \mathrm{Ni}+CO_{\left(g\right)}$$

(7) $$4\mathrm{LiM}n_{2}O_{4}\to 8\mathrm{MnO}+2Li_{2}O+3O_{2\left(g\right)}$$

(8) $$Mn_{3}O_{4}+C\to 3\mathrm{MnO}+CO_{\left(g\right)}$$

(9) $$Mn_{3}O_{4}+CO_{\left(g\right)}\to 3\mathrm{MnO}+CO_{2\left(g\right)}$$

### 2.2. Hydrometallurgical-Based Recycling of Cathode Materials

Hydrometallurgical techniques are extensively used to recover valuable metals from spent lithium-ion batteries, primarily via acid leaching, which dissolves cathode materials into ionic species using liquid solvents [37]. The leachate is then subjected to separation and purification steps such as solvent extraction, chemical precipitation, ion exchange, or electrochemical deposition. However, these approaches face challenges including complex separation steps, generation of secondary waste, and potential loss of valuable metals [55]. To maximize the utilization of leached cathode materials, regeneration processes are likely to become a key focus for future development [50]. Sattar et al. optimized process parameters such as pulp density, acid concentration, dosage of reducing agent, time, and temperature for the sulfuric acid leaching of cathode material. When leaching a cathode with 5% pulp density at 90 °C for 3 h, the maximum recovery rates reached 92% for lithium, 68% for cobalt, and 34.8% for manganese. Even at lower temperatures (e.g., 50 °C), the metal extraction rate could exceed 98% with the addition of 4 vol% hydrogen peroxide as a reductant [56]. Premanandh et al. explored a range of hydrometallurgical strategies for lithium-ion battery cathodes, analyzing how different combinations of leaching agents, purification methods, and material conversion technologies impact both environmental and economic performance. Among the evaluated technologies, deep eutectic solvent (DES) leaching emerged as the most environmentally friendly across multiple impact categories, while electrolysis-based methods offered minimal benefits. For purification processes, ion exchange-based methods showed significantly lower environmental footprints—except in the category of stratospheric ozone depletion—while solvent-based purification was assessed as the least favorable due to its high toxicity and solvent volatility. In terms of post-leaching treatment, hydroxide calcination was recognized as a more sustainable alternative compared to oxalate calcination [57].

Acid leaching can be classified into two main categories: inorganic acid leaching and organic acid leaching [44,58]. Inorganic acids—such as H_2_SO_4_, HCl, and HNO_3_—are most used and are summarized in Table 5. These strong acids effectively dissolve metal oxides; however, they may not efficiently extract less soluble, high-valence forms. To enhance the leaching of these species, reductants like H_2_O_2_ or complexing agents such as glucose are often introduced to reduce metal ions to lower oxidation states, thereby improving leaching performance [59]. Despite their strong acidity and capability to dissolve most metal ions, the industrial application of inorganic acids generates large volumes of acidic wastewater and toxic gases (e.g., Cl_2_ and NOₓ), posing significant environmental hazards [60].

In contrast, organic acid leaching systems are milder and more environmentally friendly, achieving comparable leaching efficiencies [67,68]. Common organic acids include citric acid, malic acid, glycine, and ascorbic acid, as shown in Table 6. Under low solid-to-liquid ratio conditions, organic acid leaching demonstrates satisfactory efficiency. However, due to the relatively high cost of organic acids and the immature understanding of their complex leaching mechanisms, their industrial applications remain limited. As a result, inorganic acid leaching remains the dominant approach in industrial-scale recycling [8].

### 2.3. Direct Regeneration Recycling of Cathode Materials

As an emerging strategy for LIB recycling, direct regeneration aims to restore the electrochemical performance of spent cathode materials by repairing lattice defects—such as lithium vacancies and cation mixing—without disrupting the original crystal structure. Compared to traditional pyrometallurgical and hydrometallurgical methods, direct regeneration offers substantial advantages in terms of environmental sustainability, energy efficiency, and cost-effectiveness [78]. The core mechanism involves lithium supplementation and crystal structure regulation, enabling the direct reuse of cathode materials without complex separation or purification, thereby significantly reducing energy consumption and secondary pollution [79]. This strategy has evolved into several technical pathways, encompassing high-temperature treatment, solution-phase reactions, and electrochemical restoration. Each method is defined by distinct operational parameters, target material types, and associated electrochemical performance outcomes. A summary of representative direct regeneration techniques is provided in Table 7, outlining their material targets, reaction conditions, lithium sources, and resultant electrochemical performance metrics.

Among the available techniques, the high-temperature solid-state method is one of the earliest and most established. By employing sintering under a reducing atmosphere at 600–800 °C with Li_2_CO_3_ as the lithium source, this method is particularly suitable for thermally stable materials such as LiFePO_4_ (LFP). The regenerated LFP achieved a discharge capacity of 147.3 mAh g^−1^ and maintained 95.32% of its capacity after 100 charge–discharge cycles [78]. However, its high energy demand has driven efforts to develop lower-temperature alternatives.

Hydrothermal techniques provide a milder regeneration route by utilizing ion diffusion within aqueous media. A typical protocol involves the treatment of spent LiCoO_2_ (LCO) with 4 M LiOH at 220 °C, followed by brief annealing at 800 °C, which results in a regenerated cathode with a capacity of 153.1 mAh g^−1^ and 91.2% retention after 100 cycles [78]. A more energy-efficient variant utilizes a low-temperature hydrothermal process at 80 °C, employing citric acid as a reducing agent. This method restored LFP to 159 mAh g^−1^ with a 93.7% retention rate after 100 cycles, all under ambient pressure without the need for specialized equipment [80].

Molten salt thermochemistry has demonstrated superior performance in regenerating high-nickel ternary cathode materials. The use of a LiNO_3_–LiOH eutectic salt mixture at 300 °C enabled effective repair of NCM523, achieving a discharge capacity of 149.3 mAh g^−1^ with 90.15% retention over 100 cycles [78]. This approach has been further applied to mixed cathodes such as LMO/NMC. When treated with LiNO_3_ at 300 °C for 4 h, the regenerated materials delivered 144.0 mAh g^−1^ with 95.1% retention after 250 cycles, while simultaneously avoiding the need for material separation. Additionally, this method reduced energy consumption by 90.4% and PM2.5 emissions by 51%, showcasing its environmental and economic potential [80].

Low-temperature green solvents, particularly deep eutectic solvents (DESs), have also gained attention. A LiCl–urea DES applied at 120 °C was able to regenerate spent LCO with a capacity of 133.1 mAh g^−1^ and 72.7% retention after 100 cycles. Importantly, the solvent demonstrated a high recovery efficiency of 98.7%, further underlining the method’s environmental compatibility [78].

Electrochemical methods present a unique, low-energy regeneration route by enabling lithium reinsertion through externally applied electric fields at room temperature. For example, a symmetric electrochemical setup using a Li_2_SO_4_ electrolyte, followed by annealing at 700 °C, restored LCO to 140 mAh g^−1^ with 93% retention over 100 cycles [81]. Furthermore, a redox-mediated electrochemical approach using quinone compounds such as DTBQ enabled the regeneration of NCM111 at room temperature within just 60 min, delivering an impressive initial capacity of 171 mAh g^−1^ [78]. This approach notably streamlines the regeneration process and lowers energy requirements, positioning it as a promising candidate for scalable LIB recycling.

While these laboratory-scale results are promising, scaling direct regeneration to industrial applications faces significant barriers. One primary challenge is the material heterogeneity in end-of-life LIB streams, where cathode chemistries, particle morphologies, and impurity levels vary widely. This heterogeneity can cause inconsistent lithiation efficiency and degraded electrochemical performance in regenerated products, with pilot-scale trials reporting a 10–15% lower capacity retention compared to optimized lab-scale tests when feedstock pre-sorting is absent [78]. Residual contaminants, including electrolyte decomposition products, binder residues, and transition metal cross-contamination, further hinder lithium replenishment and lattice repair, often necessitating additional cleaning or selective dismantling steps [84]. Moreover, reactor scale-up introduces heat and mass transfer limitations, particularly in molten salt and high-temperature sintering systems, leading to non-uniform lithiation. These issues have been partially addressed through hybrid strategies, such as combining molten-salt lithiation with pretreatment purification and AI-assisted feedstock classification, enabling recent pilot lines in China and South Korea to achieve > 90% capacity retention and >85% yield for mixed-NCM feedstocks [85].

### 2.4. Advanced Recycling of Cathode Materials

In contrast to the non-selective nature of acid leaching, ammonia leaching utilizes solutions such as ammonia water, ammonium carbonate, ammonium chloride, and ammonium sulfate to selectively extract transition metals via coordination interactions between NH_4_^+^ ions and metal ions [86]. This selectivity arises from differences in the solubility and stability of the coordination compounds formed between transition metals and ammonia ligands. Ammonia leaching selectively extracts valuable metals while leaving impurities behind, thereby simplifying the subsequent separation and recovery processes for metal ions from the leachate. Furthermore, combining ammonia leaching with ammonia distillation helps minimize resource waste and wastewater discharge, significantly reducing industrial costs [87]. However, in specific cases, reductants such as H_2_O_2_ and $Na_{2}S_{2}O_{5}$ are added to enhance the efficiency of ammonia leaching by reducing high-valence cobalt and nickel to Co^2+^ and Ni^2+^, respectively [88].

After extracting metal ions through leaching, the next step is to recover metals from the leachate using appropriate methods. Compared to traditional selective precipitation methods, which involve complex recovery routes and prolonged separation cycles, the regeneration method synthesizes cathode materials or precursors directly from the leachate. This approach greatly enhances the utilization of electrode materials and enables closed-loop recycling of spent lithium-ion batteries [89]. More importantly, the regenerated cathode materials retain their performance, meeting the energy density and cycling performance requirements of ternary lithium-ion batteries. Typical regeneration methods include hydrothermal methods, co-precipitation, and sol–gel methods as shown in Table 8. The hydrothermal method regenerates cathode materials through a dissolution-recrystallization mechanism. In this process, recovered cathode materials are mixed with lithium-containing solutions and heated at low temperatures (120–220 °C) in sealed reaction vessels to synthesize new cathode materials [90]. Water is used as the reaction medium to ensure uniform lithiation, and a short annealing process is typically required to enhance crystallinity [91].

The co-precipitation method is a commonly used approach for regenerating ternary cathode materials. The process begins with acid leaching of pretreated samples to obtain a solution, followed by impurity removal through precipitation or extraction [99]. The ratios of metal ions are then adjusted by introducing corresponding metal salts to achieve the desired stoichiometry. A precipitant is then introduced to co-precipitate and synthesize ternary precursors, which are mixed with lithium salts and processed via high-temperature solid-phase methods to synthesize new cathode materials [100].

The sol–gel method involves using organic acids as both leaching agents and complexing agents. A hydrolysis–polymerization reaction takes place in the leachate, producing a sol that transitions into a gel upon heating and water evaporation. The gel is then calcined to produce cathode materials [54]. The typical sol–gel process includes organic acid leaching of pretreated samples, followed by adding corresponding metal salts and ammonia to the leachate to adjust the molar ratio of metal elements and pH during the reaction. Continuous heating and stirring lead to complete water evaporation, forming a three-dimensional gel network, which is calcined to decompose organic components and obtain regenerated cathodes.

## 3. Life Cycle Assessment of Cathode Materials in Lithium Battery Recycling

Life cycle assessment (LCA) is a comprehensive analytical framework used to evaluate the environmental impacts of products throughout their entire life cycle. In the context of lithium-ion battery (LIB) recycling, LCA is essential for comparing alternative technological pathways. However, discrepancies in system boundaries, functional units, and selected impact categories across studies often hinder the comparability of results. Among various environmental indicators, global warming potential (GWP) and greenhouse gas (GHG) emissions are the most frequently assessed metrics [101]. Raugei et al. simulated lithium cobalt phosphate (LCP) batteries exhibit substantial GHG emissions during production, with a specific energy-related emission of 76.1 kg CO_2_/kWh. Similar studies on LFP, NCM, and LMO battery chemistries revealed emission intensities of 109.3, 104, and 96.6 kg CO_2_/kWh, respectively [102]. On average, producing 1 kWh of LIB energy capacity generates approximately 110 kg of CO_2_, with cathode material synthesis identified as the primary emission source—underscoring the critical role of cathode recycling in mitigating overall emissions [103].

**Figure 2:**
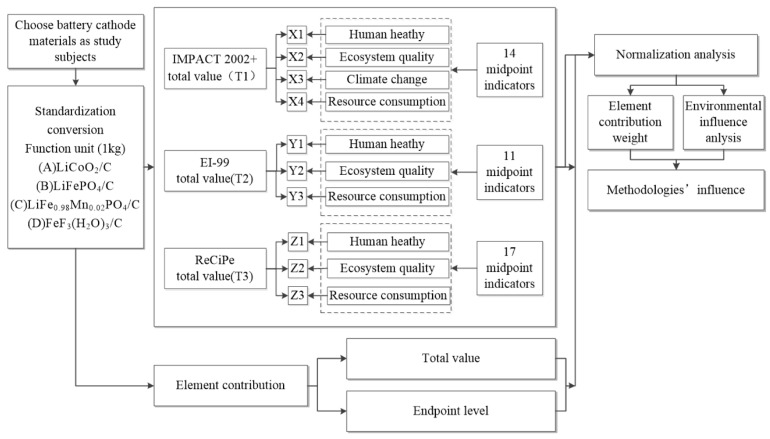
Research framework and LCA methodological process for LIB environmental sustainability assessment (adapted with permission from [105]).

LCA typically follows the ISO 14040 and 14044 frameworks, including goal and scope definition, inventory analysis, impact assessment, and interpretation. Functional units such as “1 kg battery” or “1 kWh capacity” are widely used to enable consistent comparison of environmental impacts across different systems. In terms of LCIA methodology, models such as ReCiPe, IMPACT 2002+, Eco-indicator 99, and EF 3.0 are frequently applied to translate inventory data into midpoint impact categories [104]. The general structure of LCA implementation and methodological differentiation in LIB studies is illustrated in Figure 2. The figure demonstrates how the goal and scope definition leads to specific modeling choices and impact characterization methods, including midpoint and endpoint approaches, providing a comprehensive view of how different LCA tools (such as ReCiPe and IMPACT 2002+) are integrated across assessment stages [50].

Recent LIB-focused LCA applications increasingly incorporate specialized software and databases to improve consistency, traceability, and modeling granularity. Representative tools include Simapro (v9.6) and Brightway2 for LCI and LCIA execution, with EF 3.0 and ReCiPe 2016 as prevalent impact assessment models, as shown in Table 9 [106]. Ecoinvent v3.6 or later versions are commonly used as background databases for electricity, transportation, and chemical inputs.

To bridge data gaps in emerging recycling technologies, process simulation-based LCA (PS-LCA) approaches are increasingly employed. Software such as HSC Chemistry, Aspen Plus, and SuperPro Designer facilitates thermodynamic and process-flow simulation of recycling routes, enabling the generation of primary foreground data for early-stage assessments [105].

**Figure 3:**
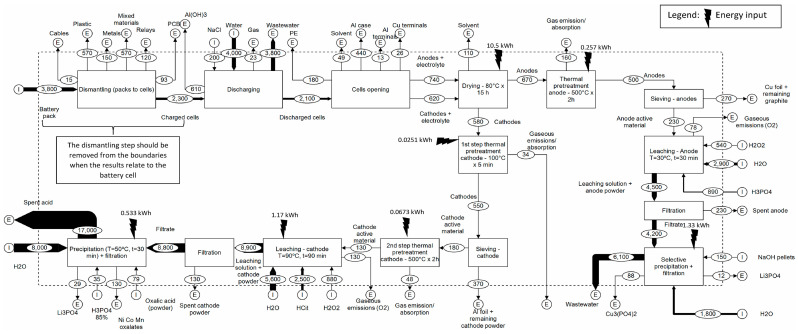
System boundaries and value chain layout for LIB production and recycling (adapted with permission from [109]).

The delineation of system boundaries is pivotal to LCA accuracy. These boundaries determine which life cycle stages—e.g., raw material extraction, manufacturing, usage, and recycling—are included in the assessment [108]. Figure 3 presents a typical system boundary configuration for LIB studies, covering both forward and reverse logistics. This system-level view is essential for evaluating closed-loop recycling systems.

Resource consumption and emissions during production and recycling phases are central to sustainability evaluations. End-of-life (EoL) recycling can offset upstream impacts from raw material extraction. For instance, hydrometallurgical treatment of LCP batteries reduces total GHG emissions by 13.6% [110]. Substituting virgin materials with recycled ones via hydrometallurgy may lower emissions by 30.91 kg CO_2_/kWh, particularly benefiting climate change and ecotoxicity categories [111]. However, the recycling process itself also entails energy consumption and environmental emissions. The actual environmental benefit thus depends on the balance between the environmental burdens of recycling and the credits gained from avoided primary material production.

To better assess and compare such trade-offs, it is essential to standardize LCA frameworks for LIB recycling. The upstream stage should quantify the number of collection points, average transport distance from collection sites to recycling facilities, and transport mode, specifying fuel type and load factor to enable consistent logistics-related emission estimates [112]. Pretreatment and material liberation processes should report energy consumption per kilogram of black mass processed, type and consumption rate of mechanical aids (e.g., shredders, sieves), and the proportion of electrode material successfully liberated [113]. The main recycling stage—whether mechanical, pyrometallurgical, hydrometallurgical, or direct regeneration—should provide comprehensive inventories detailing reagent type and concentration, reaction temperature, residence time, electricity consumption, recovery efficiency for each target metal, and the quantity of secondary waste streams generated [114]. Downstream refining to battery-grade products should specify purification methods, chemical inputs, and final product purity levels. Functional units should be standardized to both 1 kg of cathode active material and 1 kWh of battery capacity, with an additional functional unit of 1 kg black mass for pretreatment-focused studies [113]. Harmonized impact indicators, including global warming potential (kg CO_2_-eq), cumulative energy demand (MJ), and water consumption (m^3^), together with technoeconomic metrics such as processing cost (USD/kg) and net material value, will strengthen the linkage between environmental performance and economic viability [115]. Sensitivity analyses should examine variations in electricity grid mix, transport distances, and recovery efficiencies, while uncertainty analyses should address parameter ranges for reagent usage, yield rates, and logistics assumptions [115]. Adopting these quantifiable requirements will ensure boundary alignment across studies, enabling robust meta-analyses and policy-relevant comparisons of LIB recycling technologies. By ensuring consistent inclusion of upstream logistics, standardized functional units, and harmonized impact indicators, variability caused by inconsistent boundary definitions can be minimized, thereby improving the reliability and validity of cross-study comparisons.

Duarte Castro et al. compared multiple recycling strategies and found that hydrometallurgy achieves the greatest GWP reductions, while pyrometallurgy, despite its technical maturity, suffers from high energy use and emissions. Direct regeneration, although less mature, exhibits favorable environmental performance due to the preservation of material structure [109]. GHG emissions during the EoL phase range from 16 to 32 kg CO_2_/kWh, depending on the specific technology and battery chemistry [34]. Table 10 provides a comparative summary of GHG emissions and energy use for different LIB recycling processes, showing that direct recycling and advanced hydrometallurgy can significantly outperform conventional methods in both environmental and resource indicators.

Pyrometallurgy exhibits high energy consumption and significant greenhouse gas emissions due to the high-temperature processing involved. During high-temperature treatment, large quantities of harmful gases are generated, exacerbating air pollution and increasing the environmental footprint of battery recycling [105,120]. In contrast, hydrometallurgy offers relatively better environmental performance but involves complex chemical handling and waste management challenges. For instance, substantial amounts of chemical reagents are used, and the resulting wastewater requires proper treatment to prevent environmental contamination [105,120].

Among these methods, in situ reduction roasting demonstrates distinct advantages. This method has the lowest energy consumption, and its carbon dioxide emissions are significantly reduced compared to pyrometallurgy. These improvements are attributed to its lower reaction temperature and simpler process flow. However, challenges persist, such as difficulties in achieving high material purity and limited scalability for large-scale production, which hinder its broader application [118].

The recycling stage plays a pivotal role in the LCA of LFP batteries and NCM batteries. Recycling technologies for these batteries differ substantially in terms of input materials, energy consumption, output products, and pollutant emissions [111]. The environmental performance of recycling varies considerably across LIB chemistries. For LFP batteries, hydrometallurgy recovers lithium through chemical extraction, while physical recycling emphasizes mechanical separation. NCM batteries rely more heavily on hydrometallurgical and pyrometallurgical processes to recover high-value metals, although with differing energy and emissions profiles [112,116].

A comparative summary of these recycling technologies is presented in Table 11, which outlines the inputs, outputs, and emissions associated with hydrometallurgical and pyrometallurgical processes applied to LFP and NCM battery chemistries. For example, pyrometallurgical treatment of NCM batteries results in elevated CO_2_ and ammonia emissions and generates substantial quantities of residue and ash. In contrast, hydrometallurgical processes demand greater chemical and energy input—particularly in electricity and acid consumption—but produce less atmospheric pollution and allow for higher selectivity in metal recovery.

Despite technological progress, significant challenges remain in EoL battery recycling. Material heterogeneity, compositional variability, and low collection rates limit resource recovery efficiency. Moreover, upstream activities such as collection, sorting, and transportation are often excluded from LCA boundaries, despite their substantial environmental footprint. Future efforts should prioritize integrating these upstream processes into assessments and promoting standardized, energy-efficient, and low-impact recycling technologies [119].

## 4. Economic Analysis of Cathode Materials in Lithium Battery Recycling

The economic analysis of lithium-ion battery (LIB) recycling entails a comprehensive evaluation of costs and benefits across all process stages—from collection and transportation to metal refining and purification. As detailed in Table 12 summarizes the economic analysis of each recycling stage, including the necessary operations, recycling methods, and associated costs per ton. This table highlights the variability in costs across different stages, such as collection and transportation, pretreatment, material sorting and separation, and metal refining and purification.

To systematically assess environmental and economic outcomes, models such as EVERBATT and GREET are widely applied. The EVERBATT model offers a life cycle cost and impact simulation tailored to LIBs, incorporating parameters such as transport distance, recycling facility scale, and regional variation. In contrast, the GREET model evaluates energy use and quantifies GHG emissions, enabling a comparative assessment of recycled versus virgin materials, as summarized in Table 13.

A robust economic feasibility analysis typically incorporates capital expenditure (CAPEX), operational expenditure (OPEX), revenue from recovered materials, and externalities such as environmental or social benefits. Financial indicators like Net Present Value (NPV) and Cost–Benefit Ratio (CBR) are commonly used to evaluate profitability and long-term sustainability [127]. Table 14 summarizes these key components and metrics, serving as a reference framework for quantitative economic assessments in LIB recycling studies.

**Figure 4:**
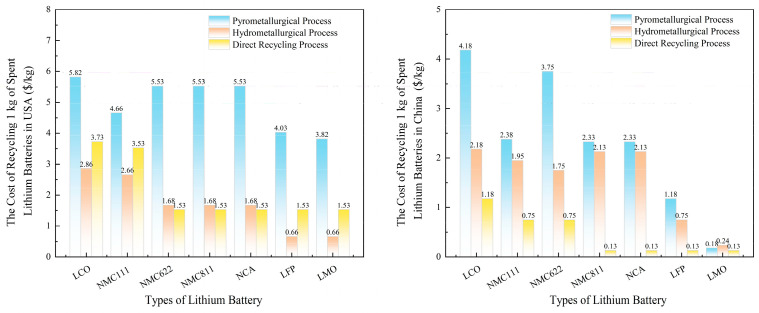
Comparison of recycling costs for spent lithium-ion batteries in the USA and China [132].

Geographic disparities significantly influence LIB recycling economics. As shown in Figure 4, recycling costs in the United States are notably higher than in China due to differences in labor wages, electricity tariffs, and reagent prices. For hydrometallurgical recycling, total costs in China are typically USD 3360–USD 4160 per ton of battery pack, while in the USA they are higher at USD 4780–USD 5640 per ton. For pyrometallurgy, reported costs are USD 3910–USD 4870 per ton in China and USD 5250–USD 6340 per ton in the USA [129]. These differences are largely attributed to labor wages. For example, battery disassembly labor costs are estimated at USD 7.50/hour in China, compared to substantially higher rates in the USA. Reagent costs are another critical factor, especially for hydrometallurgical processes. Selective leaching with sulfuric acid and hydrogen peroxide can keep reagent costs below USD 3.48/kg [130]. The availability and local production of these reagents, including sulfuric acid and hydrogen peroxide, influence regional expenses and are key cost drivers in hydrometallurgical recycling [113]. Energy costs further amplify regional disparities. Hydrometallurgy requires heating up to 95 °C, whereas pyrometallurgy operates at 1200–1450 °C, demanding far greater energy input. [131]. Additionally, recycling profitability varies across battery chemistries. LCO batteries, rich in cobalt, offer higher economic returns, whereas LFP and manganese-based batteries often yield negative net value due to lower market prices for recovered materials [16]. Simulation results show that high-value chemistries such as NMC/NCA combined with collection rates ≥90% can achieve positive margins of up to USD 1803 per ton of battery pack recycled, whereas high LFP content and low collection efficiency result in negative returns [132].

The LIB recycling process typically includes three interdependent stages: shredding and sorting, black mass handling, and cathode active material (CAM) manufacturing [133]. Among these, black mass logistics are increasingly restricted by regulatory controls—especially within the EU, where transport of hazardous materials is tightly regulated. The EU’s Critical Raw Materials (CRM) strategy prioritizes domestic retention of lithium, cobalt, and nickel, creating logistical bottlenecks for cross-border black mass transfer [134]. While European recyclers excel in mechanical pretreatment, the limited CAM manufacturing infrastructure hampers downstream processing, forcing reliance on local black mass sources that may lack sufficient volume or quality [135]. Such constraints hinder cross-border synergies, as highlighted by cases where black mass from European pretreatment facilities is unable to reach specialized hydrometallurgical plants in other regions for CAM regeneration [133]. Meanwhile, the business model adopted by stakeholders directly impacts recycling efficiency, logistics costs, and long-term economic returns. Four mainstream models have been identified based on stakeholder involvement [136].

**Figure 5:**
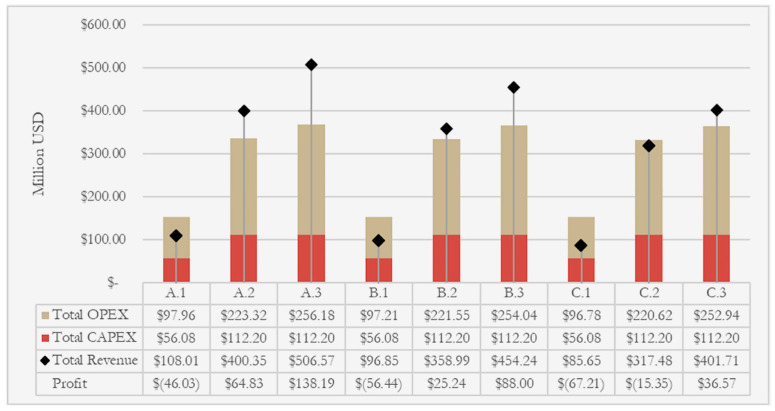
Comparison of total cost and revenue under different recycling scenarios [128].

To provide a more comprehensive understanding of the profitability dynamics across different operational strategies, Figure 5 illustrates the comparative analysis of total cost and revenue under various simulation scenarios [128]. The scenarios differ in cathode composition (e.g., high Ni/Co vs. high LFP) and waste collection rates [16]. The simulation results clearly show that scenarios involving high-value metals (such as NMC/NCA) and elevated collection rates (e.g., ≥90%) offer the highest economic returns, with positive margins up to USD 1803 per ton of battery pack recycled. Conversely, scenarios with high LFP content and low collection efficiency result in negative returns, even when processing costs are minimized [137]. Among the cost components, reagent usage in the hydrometallurgical stage consistently dominates total operating expenses, indicating the critical importance of process optimization.

**Figure 6:**
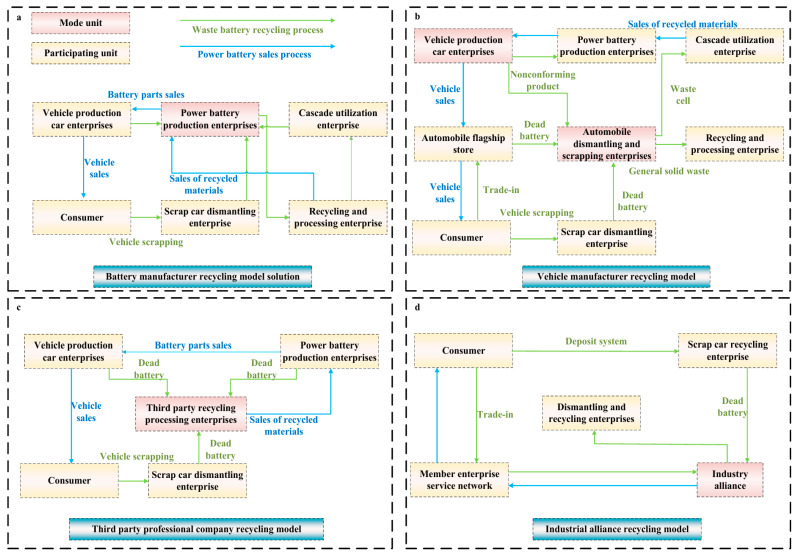
The current recycling logistics of battery. (**a**) Battery manufacturer recycling model solution, where battery production enterprises take the lead in the recycling process; (**b**) Vehicle manufacturer recycling model, in which vehicle production companies manage battery recovery by leveraging their after-sales service systems; (**c**) Third party professional company recycling model, relying on specialized third-party enterprises to conduct recycling and processing; and (**d**) Industrial alliance recycling model, coordinated by an industrial alliance to realize battery recycling through collaborative networks.

The battery manufacturer-led recycling model positions battery manufacturers as the primary entities responsible for collecting and recycling end-of-life batteries as shown in Figure 6a. Various business models have been adopted to improve the economic viability and operational efficiency of LIB cathode material recycling. One common approach is the battery manufacturer-led model, where producers such as CATL and BYD oversee the entire recycling chain through subsidiaries or strategic partnerships. This model promotes a stable supply of secondary materials and supports closed-loop production systems, though it may be constrained by limited collection channels and significant upfront capital investment requirements [37].

Another approach is the vehicle manufacturers-led model, in which automakers like Tesla, Toyota, and Mercedes-Benz take charge of battery recovery by utilizing their after-sales service networks, such as 4S dealerships and authorized repair shops. This method is cost-effective and offers high efficiency through integrated logistics and data-driven recycling optimization, but is often confined to batteries from the automaker’s own brand, limiting its broader scalability, as shown in Figure 6b [138].

A third model is led by third-party professional recycling companies such as GEM, Huayou Cobalt, and Brunp. These enterprises possess mature recycling technologies and specialized processing capabilities. While their operations are typically highly professionalized, they also involve high infrastructure investment and logistical complexity, particularly in terms of transporting and reselling recovered materials, as shown in Figure 6c [139].

Figure 6d illustrates the industry alliance-based model brings together multiple stakeholders—battery manufacturers, electric vehicle producers, and recycling enterprises—into cooperative networks. By sharing infrastructure, expertise, and logistics, this collaborative framework enhances resource utilization and recycling efficiency. However, it demands strong inter-organizational coordination and remains at an early stage of deployment in most regions [140].

## 5. Conclusions

Efficient recycling of spent lithium-ion battery (LIB) cathode materials is pivotal to supporting the global shift toward low-carbon energy systems and mitigating resource scarcity. This review presents a comprehensive evaluation of both established and emerging recovery strategies, integrating technological comparisons, economic feasibility, and business model evaluations. Mature processes such as pyrometallurgy and hydrometallurgy remain dominant due to their scalability and high recovery efficiency. However, they pose trade-offs in terms of energy intensity, emissions, and chemical waste generation. Emerging approaches—including organic acids, deep eutectic solvents (DESs), and in situ roasting—offer improved environmental profiles but demonstrate improved environmental performance but require further industrial validation. Among these, direct regeneration techniques, which enable the direct reuse of cathode materials by repairing their crystal structures, show promise for promoting material circularity. Regeneration techniques, which enable direct reuse of cathode materials, represent a promising pathway for achieving material circularity. Integrated life cycle and technoeconomic analyses reveal that the profitability of LIB recycling is highly sensitive to cathode chemistry, collection efficiency, and reagent usage—particularly in hydrometallurgical systems where reagents are a major cost driver. Simulation-based studies confirm that recycling scenarios involving high-value chemistries (e.g., NMC/NCA), combined with efficient logistics and high collection rates, offer both environmental and economic benefits. Moreover, the choice of business model significantly affects the economic and operational performance of recycling systems. Manufacturer-led and alliance-based models can tend to enhance collection efficiency and supply chain integration, while third-party recycling systems provide technical expertise and specialization. Despite recent progress, several barriers remain, including inconsistent system boundaries, uneven collection infrastructure, and regional cost disparities. To ensure the scalability and sustainability of LIB recycling systems, future research and policy should focus on the development of harmonized evaluation frameworks, the innovation of green and cost-effective reagents, and the optimization of upstream value chains. Cross-sector collaboration between science, industry, and government is critical to advancing economically viable, environmentally responsible, and globally applicable LIB recycling solutions.

## Figures and Tables

**Table 1:** Economic value prediction of metal recycling from spent ternary lithium batteries (NCM) [15,16].

Recycling Product	Recovery Rate	Market Price (USD/kg)
Cu	-	7.54
Ni	95–98%	21.72
Co	95–98%	46.3
Mn	-	0.01
Li	90–95%	62.26

**Table 2:** Recycling technologies and main recycled products of diverse spent battery enterprises.

Company	Location	Technology	Main Products	Ref.
Accurec	Germany	Pyrometallurgy and Hydrometallurgy	Co alloy, Li_2_CO_3_	[18]
Batrec	Switzerland	Pyrometallurgy and Hydrometallurgy	Battery scraps	[19]
Duesenfeld	Germany	Mechanical treatment and Hydrometallurgy	Co, Li salt	[20]
GEM	China	Hydrometallurgy	Co, Ni powder, Ni/Co alloy, Co_3_O_4_	[21]
Glencore (former Xstrata)	Switzerland	Pyrometallurgy and Hydrometallurgy	Alloy (Co/Ni/Cu)	[22]
Inmetco	USA	Pyrometallurgy and Hydrometallurgy	Co, Ni, and Fe alloy	[23]
OnTo Technology	USA	Mechanical treatment and Hydrometallurgy	Cathode powder	[24]
Recupyl	France	Hydrometallurgy	Co(OH)_2_ and Li_2_PO_4_, Li_2_CO_3_	[25]
Retriev Technologies & Toxco (Canada)	USA and Canada	Hydrometallurgy	CoO, Li_2_CO_3_, mixed metal oxides	[26]
Sumitomo-Sony	Japan	Pyrometallurgy and Hydrometallurgy	CoO	[27]
Umicore	Belgium	Pyrometallurgy and Hydrometallurgy	Co salt, Ni salt, Cu salt, mixed metal oxides	[28]

**Table 3:** Lithium-ion battery cathode material recycling process and technology characteristics.

Recycling Stage	Technology Type	Representative Methods	Main Products	Advantages	Disadvantages
Pretreatment	Battery Discharge	NaCl solution immersion, Freezing	Discharged batteries	Safe deactivation	Limited to early-stage processing
Mechanical Processing	Physical Separation	Crushing, Screening, Manual dismantling	Al, Cu foil, shell, active material	Simple operation, high separation efficiency	High energy use, gas/solvent emissions
Cathode Recovery	Pyrometallurgy	Reduction smelting, Carbothermal, Roasting	Slag, metal ion filtrate	Simple, scalable, high metal tolerance	High temperature, lithium loss, gas emissions
	Hydrometallurgy	Inorganic/organic acid, ammonia leaching	Metal ion filtrate	High efficiency, low energy, flexible conditions	Wastewater, toxic gas risks
Metal Refining	Separation and Purification	Solvent extraction, Precipitation, Sol–gel	Purified metals or compounds	High purity, high recovery	Complex process, variable product stability

**Table 4:** An overview of published pyrometallurgical recycling approaches for lithium-ion batteries.

Process	Temp.	Additive	Pretreatment	Conditions	Separated Materials	Secondary Treatment	Recovery Rate	Ref.
Reduction Roasting	650 °C	Carbon	Not specifically mentioned	650 °C, 30 min	Li_2_CO_3_, Co, Ni, NiO, MnO	Water and acid leaching (H_2_SO_4_)	Li: 93.67%, Ni: 93.33%, Co: 98.08%, Mn: 98.68%	[41]
Sulfation Roasting	700 °C	SO_2_(g)	Not specifically mentioned	700 °C, 120 min	Li_2_SO_4_, Li_2_Co(SO_4_)_2_, CoO	Water leaching	Li: 99.5%, Co: 17.4%	[42]
Sulfation Roasting	750 °C	Na_2_SO_4_	NaCl immersion, Manual dismantling, Calcination	750 °C, 90 min	Li_2_SO_4_, MnO, NiO, CoO, CuO_2_	Water leaching	Li: 85.43%, Ni, Co, Mn all 84.93%	[43]
Nitration Roasting	250 °C	HNO_3_	Mechanical pretreatment	250 °C, 60 min	LiNO_3_, Co(NO_3_)_2_,	Water leaching	Li: 93%, Co, Ni, Cu all 92.9%	[44]
Vacuum Pyrolysis	600 °C	Carbon	NaCl discharging, Manual dismantling, Vacuum pyrolysis	600 °C	Co, CoO, Li_2_CO_3_	Water leaching	Li: 93%, Co: 99%	[45]
Chlorination Calcination	350 °C	NH_4_Cl	Discharging, Manual dismantling, NaOH dissolution	350 °C for 20 min	LiCl, CoCl_2_,	Water leaching	Li: 99.18%, Co: 99.3%	[46]
Microwave Carbothermic Reduction	900 °C	Carbon	NaCl discharge, Manual dismantling, comminution	900 °C, 500 W, 30 min	Not specifically mentioned	Acid leaching (HCl(aq))	Li: 99.68%, Ni: 97.65%, Co: 97.85%, Mn: 96.73%	[47]
Carbothermic Reduction Smelting	1450 °C	Cu slag (slag former)	Not specifically mentioned	1450 °C for 30 min	Co, Ni, Cu and Fe alloy and slag	Manual separation of slag and alloy, comminution	Co: 98.83%, Ni: 98.39%, Cu: 93.57%	[48]
Reduction Smelting	1475 °C	Pyrolusite slag former, SiO_2_, CaO	Roasting at 800 °C for 120 min to remove carbon	1475 °C, 30 min	Co-Ni-Cu-Fe alloy	Manual separation, comminution, acid leaching	Li: 79.86%, Mn: 94.85%	[49]

**Table 5:** Inorganic leaching processes for recycling spent lithium-ion batteries.

Inorganic Leaching Agent	Reducing Agent	Temp. (°C)	Time	Efficiency (%)			Ref.
				Li	Co	Mn	
$H_{2}SO_{4}\;({2.0\;\mathrm{mol}\cdot L\;}^{-1})$	$H_{2}O_{2}\;(3.0\;\mathrm{Vol}\%)$	60	-	98.9	98.4	98.6	[61]
$H_{2}SO_{4}\;({0.2\;\mathrm{mol}\cdot L\;}^{-1})$	Citric Acid ${(0.05\;\mathrm{mol}\cdot L\;}^{-1})$	120	2 h	99.5	99.5	99.7	[62]
$H_{2}SO_{4}\;({3.0\;\mathrm{mol}\cdot L\;}^{-1})$	Glucose (16.0 Vol%)	90	2 h	99.54	99.58	99.1	[63]
HCl $\left({3\;\mathrm{mol}\cdot L\;}^{-1}\right)$	$H_{2}O_{2}\;({0.3\;\mathrm{mol}\cdot L\;}^{-1})$	80	1.5 h	99.4	-	-	[64]
$H_{3}PO_{4}\;({0.7\;\mathrm{mol}\cdot L\;}^{-1})$	$H_{2}O_{2}\;(4.0\;\mathrm{Vol}\%)$	40	1 h	99	99	-	[65]
$H_{3}PO_{4}\;({0.2\;\mathrm{mol}\cdot L\;}^{-1})$	Citric Acid (0.4 mol/)	90	0.5 h	100	91.63	92	[66]

**Table 6:** Organic leaching processes for recycling spent lithium-ion batteries.

Inorganic Leaching Agent	Reducing Agent	Temp. (°C)	Time	Efficiency (%)			Ref.
				Li	Co	Mn	
DL-malic Acid (1.5 mol/L)	$H_{2}O_{2}\;(3.0\;\mathrm{Vol}\%)$	80	25 min	98.13	98.86	-	[69]
DL-malic Acid (1.25 mol/L)	Glucose $\left({0.3\;\mathrm{mol}\cdot L\;}^{-1}\right)$	80	3 h	100	99.87	-	[70]
Citric acid $\left({1.5\;\mathrm{mol}\cdot L\;}^{-1}\right)$	$H_{2}O_{2}\;(2.0\;\mathrm{Vol}\%)$	95	20 min	96	90	94	[71]
Citric acid $\left({2\;\mathrm{mol}\cdot L\;}^{-1}\right)$	Cu	70	24 h	97.8	81.3	-	[72]
Glycine $({1.5\;\mathrm{mol}\cdot L\;}^{-1}$)	$Na_{2}S_{2}O_{5}\;({1.5\;\mathrm{mol}\cdot L\;}^{-1})$	80	3 h	99.8	100	-	[73]
Glycine $({4\;\mathrm{mol}\cdot L\;}^{-1}$)	$H_{2}O_{2}\;(10.0\;\mathrm{Vol}\%)$	80	7 h	90.95	97.07	-	[74]
Ascorbic acid (1.25 mol/L)	-	70	20 min	98.5	94.8	-	[67]
Ascorbic acid (2 mol/L)	-	75	1.5 h	96.3	94.8	95.6	[75]
Oxalic acid (1 mol/L)	-	95	2.5 h	98	97	-	[76]
Oxalic acid (0.5 mol/L)	-	80	20 min	95.7	-	-	[77]

**Table 7:** Comparison of direct regeneration methods for spent lithium-ion battery cathodes.

Method Type	Treated Material	Temperature	Lithium Source/Medium	Reaction Time	Post-Treatment	Regenerated Performance (Initial Capacity/Cycle Retention)	Ref.
High-temperature solid-state method	LFP	600–800 °C	$Li_{2}CO_{3}$	-	Sintering in reductive atmosphere	147.3 mAh g^−1^/95.32% (after 100 cycles)	[78]
Hydrothermal method	LCO	220 °C	4 M LiOH solution	4 h	Annealing at 800 °C	153.1 mAh g^−1^/91.2% (after 100 cycles)	[78]
Molten salt thermochemistry	NCM523	300 °C	LiNO_3_-LiOH eutectic salt	4 h	Annealing at 850 °C	149.3 mAh g^−1^/90.15% (after 100 cycles)	[78]
Low-temperature sintering method	LMO/NMC mixture cathode	300 °C	$L\mathrm{iN}O_{3}$	4 h	-	144.0 mAh g^−1^/95.1% (after 250 cycles); 83 mAh g^−1^ at 2 C	[80]
Deep eutectic solvent (DES) method	LCO	120 °C	LiCl–urea DES	4 h	Annealing at 850 °C	133.1 mAh g^−1^/72.7% (after 100 cycles); DES recovery rate 98.7%	[78]
Electrochemical method	LCO	Room temperature	$Li_{2}{\mathrm{SO}}_{4}$ solution	-	Annealing at 700 °C for 6 h	140 mAh g^−1^/93% (after 100 cycles)	[81]
Chemical lithiation method	LFP	Room temperature	Pyrene (reducing agent + lithium source)	10 min	-	Repaired Li vacancies, restored Fe^2+^ valence state, improved cycle stability	[82]
Low-temperature hydrothermal method (Co-free)	LFP	80 °C	LiOH solution + citric acid (reducing agent)	-	Ambient pressure operation	159 mAh g^−1^/93.7% (after 100 cycles)	[83]

**Table 8:** Typical regeneration methods for spent lithium-ion batteries.

Method	Reductant and Lithium Source	Temp.	Time	Electrochemical Performance	Technical Readiness Level (TRL)	Capital Expenditure (CAPEX)	Operational Expenditure (OPEX)	Practical Implementation Barriers	Ref.
One-step hydrothermal method	N_2_H_4_·H_2_O + Li_2_SO_4_·H_2_O	200 °C	3 h	146.2 mAh/g (0.2 C), 128.2 mAh/g (5 C); 98.6% after 200 cycles	3–4 (Lab-scale validation)	Lower: Relies on hydrothermal autoclaves, filtration, and drying units; no complex wastewater treatment systems required	Lower: Main costs from Li_2_SO_4_·H_2_O (lithium source), N_2_H_4_·H_2_O (reductant), and low energy consumption; total ~USD 1130/ton (22.2% of LFP price)	1. Controlling the uniformity of temperature and pressure. 2. Residual impurities (carbon black and PVDF)	[92]
	DL-malic acid + LiOH·H_2_O	100 °C	6 h	138.4 → 136.6 mAh/g @1 C (200 cycles); CE > 97.2%	3 (Lab-scale validation)	Lower: Similarly to above, using standard hydrothermal equipment; no toxic waste treatment required	Lower: DL-malic acid is low cost (~USD 2.1/kg, cheaper than N_2_H_4_·H_2_O); LiOH·H_2_O as Li source; energy consumption reduced due to lower temperature		[93]
	Tartaric acid + LiOH·H_2_O	200 °C	3 h	165.9 → 114.96 mAh/g (0.1 C–5 C); 99.1% after 200 cycles	3 (Lab-scale validation)	Lower: Standard hydrothermal setup, no hazardous waste treatment	Lower: Uses green reductants, reducing chemical costs and environmental impact		[94]
Co-precipitation	H_2_O_2_/Na_2_CO_3_ + Li_2_CO_3_, LiOH·H_2_O	25 °C	—	239.4 mAh/g (0.1 C); 81.0% retention (100 cycles); 105.1 mAh/g @5 C	3–4 (Lab-scale validation)	Moderate: Requires co-precipitation reactors, sintering furnaces, and purification systems; higher than hydrothermal methods but lower than hydrometallurgy	Moderate: Costs include leaching reagents (H_2_SO_4_, H_2_O_2_), Li_2_CO_3_, and energy for sintering; lower chemical consumption than hydrometallurgy	1. Temperature affects crystallinity/loss 2. Diffusion causes segregation/mixing	[95,96]
Sol–Gel	H_2_O_2_ + CH_3_COOLi·2H_2_O	70 °C (leaching), 900 °C (sintering)	60 min (leaching), 12 h (sintering)	NCM-Ma: 151.6 mAh/g (0.2 C), 120.2 mAh/g (5 C); 84% retention after 150 cycles at 0.2 C	3–4 (Lab-scale validation)	Moderate: Leaching tanks, sol–gel reactors, and sintering equipment; similar to co-precipitation	Lower than hydrometallurgy: Maleic acid (~USD 2.1/kg) and H_2_O_2_ costs are lower than inorganic acids; energy dominated by sintering	Gel uniformity sensitive to conditions	[97]
	Acetic/maleic acid + acetate salts	70 °C (leaching), 900 °C (sintering)	20 min (leaching), 12 h (sintering)	R-NCM: 138.2 mAh/g (0.5 C) after 100 cycles, 96% retention; 120.6 mAh/g (5 C)	3–4 (Lab-scale validation)	Moderate: Similarly to maleic acid process; leaching and sol–gel equipment	Lower: Lactic acid is biodegradable and low cost; H_2_O_2_ usage (0.5 vol%) minimizes reductant costs; short leaching		[98]

**Table 9:** LCA software and database tools for LIB recycling.

Tool	Type	Application Stage	Key Features	Ref.
Simapro v9.6	Commercial LCA software	LCIA phase	Supports EF 3.0, ReCiPe, Eco-Indicator; widely used in EU and Asia	[106]
HSC Chemistry v10.5	Process simulator	Foreground data modeling	Thermodynamic simulation for recovery routes	[106]
GaBi	Commercial LCA software	LCA modeling and impact assessment	Used for attributional LCA modeling, integrates with Ecoinvent database for background processes	[105]
Ecoinvent v3.8	LCI database	Background data	Power grid mix, logistics, chemical reagents	[107]

**Table 10:** Environmental performance of spent lithium-ion batteries (SLIBs) recycling methods.

Initial Feeding	Recycling Step	Metal Recovery	Energy Consumption	System Boundaries	Functional Units	Impact Categories	GHG Emissions	Ref.
SLIBs/NMC	Hydro/solvent extraction	Ni 92.4%, Co 92.3%, Mn 30.1%, Li 89.3%	–	Grave-to-gate: upstream (collection, transportation, dismantling), midstream (hydrometallurgical recycling), downstream (precursor CAM synthesis, calcination)	1 kg of cathode active material (CAM)	Global Warming Potential (GWP, kg CO_2_e)	6.46 kgCO_2_/kg CAM	[112]
SLIBs/NMC&NCA	Hydro	–	–	Cradle-to-gate: mineral acquisition, cell production, module/pack assembly, end-of-life treatment (excludes use phase and transportation)	1 kgCO_2_eq per kWh of battery capacity	Global Warming Potential (GWP100, kgCO_2_eq)	49.2 kgCO_2_/kWh	[116]
	Direct Recycling						65.9 kgCO_2_/kWh	
	Pyro						70 kgCO_2_/kWh	
SLIBs	Shredding-hydro/precipitation	Co 97%, Mn 98%, Li 80%	–	Shredding, leaching, precipitation (focus on wet processing stage)	1 kg of input electrode material	Resource recovery efficiency, chemical usage impact, waste generation from shredding	–	[113]
SLIBs	Shredding-hydro/solvent extraction	Li 85%, Ni 97%, Mn 99%, Co 98%		Shredding, acid leaching, solvent extraction (includes solvent regeneration)		Solvent recyclability, metal purity, energy for extraction processes		
SLIBs	Disassembly hydro/calcination	NMC 95%, LMO 95%, Al 100%		Disassembly, delamination, calcination (excludes manual disassembly labor costs)		Material purity, thermal energy for calcination, reduction in cross-contamination		
SLIBs	Hydro	–	160.7 MJ/kg battery	Cradle-to-gate for recycling: leaching, solvent extraction, precipitation (excludes upstream mining and battery production)	1 kg of battery	Global Warming Potential (GWP, kgCO_2_/kg battery), energy consumption (MJ/kg battery)	10.811 kgCO_2_/kg battery	[117]
SLIBs	Pyro		152.5 MJ/kg battery				11.342 kgCO_2_/kg battery	
SLIBs	Pyro	–	0.0536 (kwh/t) + 35.68 (kwh/t)	Cradle-to-gate for recycling: collection, transportation, pretreatment (dismantling), cascade utilization (remanufacturing), recovery utilization (smelting)	1 ton of spent LIBs	Global Warming Potential (GWP, kgCO_2_-eq/t), energy consumption (kWh/t, diesel L/t)	713.3 kgCO_2_/t	[115]
SLIBs/NMC	Direct Recycling	–	–	Recycling (supercritical CO_2_ extraction, relithiation), cell remanufacturing	1 kg of battery	Global Warming Potential (GWP), water consumption	29.27 kgCO_2_/t	[118]
SLIBs/NCM111	Hydro	Cu 70%, Al 70%, Co 90%, Ni 90%, Mn 90%	149.80 MJ/kg battery	Recycling (discharge, disassembly, shredding, leaching), cathode remanufacturing, cell remanufacturing (excludes collection and transportation)	1 kg of battery cell	Energy consumption (MJ/kg), Global Warming Potential (GWP, kgCO_2_eq/kg)	10.53 kgCO_2_/kg	[119]
SLIBs/NMC&NCA	Ultra-High Temperature (UHT)	Li and Co; 284.2 kg of LCO per FU	43 MJ/kg LCO	Collection and transportation, pretreatment (discharge, crushing), UHT smelting, hydrometallurgical purification, regeneration	1 ton of spent EV LIBs	Energy consumption (MJ/t), Global Warming Potential (GWP, kgCO_2_-eq/t)	1371 kgCO_2_-eq/t	[105,120]
	Hydrometallurgy	Co, Li, etc.;	19,637 MJ/FU			Energy consumption (MJ/t), GWP, acidification potential (AP)	1393 kgCO_2_-eq/t	

**Table 11:** Inventory data for a comparison of recycling technologies for LFP and NCM batteries [121].

Battery Type	Technology	Main Inputs	Energy Input	Main Product	Atmospheric Emissions	Waste
LFP	Hydrometallurgy	EoL LFP, HCl, H_2_O_2_, NaOH, CaCl, Water	Electricity: 0.485 kWh	LiCl (solid)	Dust, HF, VOCs, HCl mist	Plastic, etc.
NCM Battery	Hydrometallurgy	EoL NCM, H_2_SO_4_, HCl, Na_2_CO_3_, NH_3_, H_2_O_2_, Li_2_CO_3_	Gas: 0.28 m^3^	NCM cathode material	Ni, CO_2_	Wastewater, ammonia-N, etc.
	Pyrometallurgy	EoL NCM, H_2_SO_4_, Na_2_CO_3_, NH_3_, Li_2_CO_3_	Coal: 0.93 kg	NCM cathode material	Ni, CO_2_	Wastewater, ammonia, etc.

**Table 12:** Economic analysis summary of each recycling stage for spent lithium-ion batteries.

Recovery Stage	Necessary Operations	Recycle Method	Cost (USD/ton)	Ref.
Collection and Transportation	Retrieval from EV stations, retail sites; transport to central hubs	-	5–15	[112]
Pretreatment	Discharge, disassembly, crushing, casing removal	-	10–20	[122]
Material Sorting and Separation	Extraction of valuable metals (Co, Ni, Li, Mn)	Pyrometallurgy	200–300	[123]
		Hydrometallurgy	150–250	[124]
Metal Refining and Purification	Final refinement to achieve battery-grade purity	-	30–80	[125]

**Table 13:** Models for assessing the economics of lithium-ion battery recycling [126].

Model Name	Developer	Key Features	Application for Lithium Battery Recycling	Example of Application	Limitations
EVERBATT	Argonne National Laboratory, USA	Full life cycle modeling, cost estimation, environmental impact evaluation	Scenario comparison for LIB recycling	Lacks transparency in some parameters	Full life cycle modeling, cost estimation, environmental impact evaluation
GREET		Focuses on energy-related emissions, less on cost	GHG analysis of recycling vs. mining	Does not evaluate cost structures	Focuses on energy-related emissions, less on cost

**Table 14:** Summary of economic evaluation elements and metrics for LIB recycling [127,128].

Category	Element	Description
Direct Cost	CAPEX (Capital Expenditure)	Initial investments in equipment, facilities, transport fleet
	OPEX (Operational Expenditure)	Labor, utilities (electricity, water), reagents (e.g., H_2_SO_4_, NaOH), maintenance
Revenue	Material Recovery Value	Income from resale of Li_2_CO_3_, Co(OH)_2_, Ni(OH)_2_, Cu, Al
Environmental and Social	Environmental Benefits	Emission reduction, energy savings, landfill reduction
	Social Benefits	Employment creation, regional economic stimulation
Financial Indicators	NPV (Net Present Value)	Discounted future net cash flows of the project
	CBR (Cost–Benefit Ratio)	Ratio of total benefits to total costs
	RMSE, AE, R^2^ (from ML models)	Used in data-driven prediction of economic returns
